# Peak Procedural ACT Is Associated With All-Cause Mortality After Femoral Access PCI

**DOI:** 10.1016/j.jscai.2024.102387

**Published:** 2024-09-23

**Authors:** Revathy Sampath-Kumar, Ori Ben-Yehuda, Belal Al Khiami, Lawrence Ang, Anna Melendez, Ryan Reeves, Ehtisham Mahmud

**Affiliations:** Division of Cardiovascular Medicine, Sulpizio Cardiovascular Center, University of California San Diego, San Diego, California

**Keywords:** bleeding, maximal activated clotting time, percutaneous coronary intervention mortality, transfemoral, transradial

## Abstract

**Background:**

A minimum threshold activated clotting time (ACT) to guide heparin dosing during percutaneous coronary intervention (PCI) is associated with lower ischemic complications. However, data are variable regarding the risk of high ACT levels. The aim of this study was to assess the impact of peak procedural ACT on complications and mortality for transfemoral and transradial access PCI.

**Methods:**

The UC San Diego Health National Cardiovascular Data Registry CathPCI Registry was used to obtain data on patients who underwent native vessel PCI from January 2007 to September 2022. Coronary artery bypass graft patients and those who received bivalirudin were excluded. Complications and all-cause mortality at 30 days and 1-year post-PCI were assessed by ACT tertile.

**Results:**

A total of 2473 patients (age 65 ± 12 years; 74% male) undergoing PCI with 53% femoral and 47% radial access were included. The majority (82%) had 1-vessel coronary artery disease with heterogeneous clinical presentations (21.8% ST-elevation myocardial infarction, 25.4% non–ST-elevation myocardial infarction, 4.9% unstable angina, 33.8% stable angina, 3.4% atypical chest pain, 10.7% other indication for PCI). With femoral access, patients in the third tertile (ACT ≥ 275) had significantly higher all-cause mortality at 30 days (5.3% vs 2.7% vs 0.9%; *P* < .001), 6 months (6.3% vs 4.0% vs 2.0%; *P* = .007), and 1 year (9.0% vs 6.0% vs 2.7%; *P* < .001) compared to the second (ACT 228-275) and first tertile (ACT ≤ 228), respectively. A 30-day landmark analysis revealed that there was no difference in all-cause mortality beyond 30 days (3.9% vs 3.4% vs 1.8%; *P* = .176). There were increased bleeding complications in the highest tertile (12.8% vs 9.8% vs 7.5%; *P* = .034) and a higher need for blood products (10.4% vs 6.7% vs 5.4%; *P* = .014). There was no difference in ischemic major adverse cardiovascular events specifically periprocedural myocardial infarction or stroke between tertiles. There was no difference in clinical outcomes by peak ACT for patients who had radial access.

**Conclusions:**

Higher ACT with transfemoral access PCI was associated with increased 30-day mortality, bleeding complications, and need for blood products post-PCI.

## Introduction

Unfractionated heparin (UFH) has been the primary anticoagulant in percutaneous coronary intervention (PCI) for over 50 years and is used to prevent ischemic complications and optimize angiographic results. Due to the lower cost and ability for reversal, it remains the most widely used anticoagulant in the modern complex PCI era. UFH has a narrow therapeutic window with variable anticoagulant effects based on weight, renal function, and cellular factors, and thus monitoring with activated clotting time (ACT) is recommended.^1^

Current practice guidelines^2^ recommend achieving an ACT of 250 to 300 seconds using the HemoTec (Hemotec Inc) or I-Stat (Abbott) devices and an ACT of 300 to 350 seconds using the Hemochron ACT (Werfen) devices with consideration of higher target ACT in the setting of chronic total occlusion or acute coronary syndrome. If glycoprotein IIb/IIIa inhibitor (GPI) use is planned, a lower target ACT of 200 to 250 seconds is recommended.

Activated clotting time cutoffs to guide heparin dosing during PCI were established from early retrospective studies with conflicting results relating ACT to clinical outcomes. Several studies have shown that lower ACT is associated with thrombotic complications.^3^, ^4^, ^5^, ^6^, ^7^, ^8^, ^9^ The majority of these studies were published prior to the modern coronary stent era and the widespread pretreatment with potent P2Y12 inhibitors. Additional studies found no association between lower ACT and thrombotic events^10^, ^11^, ^12^, ^13^ whereas one study paradoxically found higher thrombotic complications with higher ACT.^14^ ACT measurement may also vary by sample site; a recent study found lower ACT values from the arterial sheath compared to the guide catheter.^15^

Higher ACT has been associated with major^5^^,^^7^^,^^10^^,^^14^ and minor^11^^,^^16^ bleeding complications in both transfemoral (TF) and transradial (TR) PCI but most studies did not separate outcomes by access site. More recently, Louis et al^13^ showed that higher ACT is only associated with major bleeding in TF PCI but not TR PCI, and only reported in-hospital outcomes. Other studies found no association between ACT and bleeding complications,^4^^,^^6^^,^^8^^,^^9^^,^^16^, ^17^, ^18^ most recently in a cohort of patients with greater than 90% radial access.^12^ Access site may be an important factor when evaluating the bleeding risk associated with peak ACT.

Although no study has found a relationship between ACT and mortality or major adverse cardiovascular events (MACE),^9^^,^^12^^,^^13^^,^^16^ the majority of these studies only evaluated in-hospital and 30-day outcomes. Meta-analyses with pooled data have been inconclusive^19^ or found no association with MACE or major bleeding.^18^ The aim of this study was to determine the impact of peak procedural ACT for patients undergoing native vessel PCI with UFH as the procedural anticoagulant, on bleeding complications, ischemic MACE, and all-cause mortality up to 1-year post-PCI.

## Methods

### Data source

The UC San Diego Health National Cardiovascular Data Registry (NCDR) CathPCI Registry was used to obtain data on patients who underwent PCI from January 2007 to September 2022 at UC San Diego health sites in La Jolla or Hillcrest. UC San Diego is an academic tertiary and quaternary referral hospital system that serves as a primary PCI and ST-elevation myocardial infarction (STEMI) receiving center for a diverse group of patients from San Diego County and Imperial County, California, and from Mexico. PCI procedures were performed by attending interventional cardiologists with or without interventional cardiology fellows.

Institutional patient and procedure level data were extracted from the electronic NCDR CathPCI Registry. The NCDR CathPCI Registry data were merged with peak ACT measurements from the PCI procedure log. Target ACT was determined by the physician operator at the time of the procedure. ACT was measured using the Hemochron ACT device with blood samples generally taken from the guide catheter. Bleeding complications recorded in the NCDR CathPCI Registry were adjudicated using the electronic medical record. All-cause mortality up to 1-year post-PCI was extracted by the Health Information Management team at UCSD from the electronic medical record and confirmed by the California Department of Public Health vital records and decedent records registry. The Institutional Review Board of the University of California, San Diego approved the study (#809443).

### Patient selection

**Figure 1:**
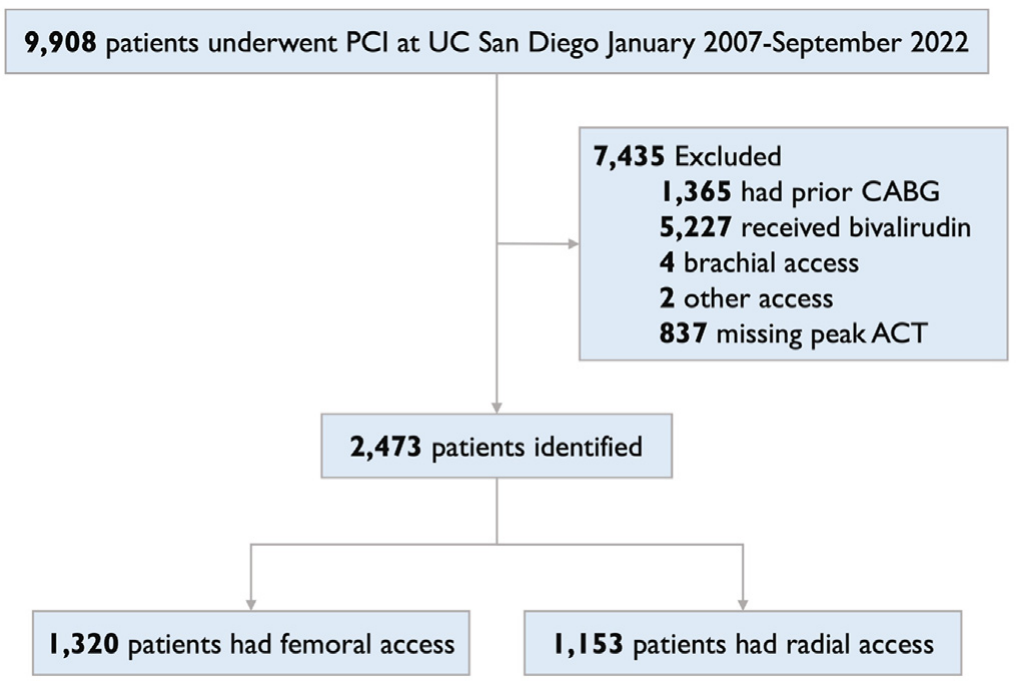
**Consolidated Standards of Reporting Trials(CONSORT)diagram of patients included in the study.** ACT, activated clotting time; CABG, coronary artery bypass graft; PCI, percutaneous coronary intervention.

A total of 9908 adult patients underwent PCI at UC San Diego Health from January 2007 to September 2022. Among these patients, 1365 patients had coronary artery bypass grafts and were excluded while 5227 patients received bivalirudin and were excluded. Patients undergoing PCI with brachial or alternative access (n = 6) were excluded. In 837 patients, a peak ACT was not obtained, and therefore, 2473 patients were included in the final cohort (1320 with TF access and 1153 with TR access) (Figure 1). This study followed the Consolidated Standards of Reporting Trials (CONSORT) reporting guideline.

### End points and definitions

Peak ACT tertiles were calculated for the entire population (N = 2473) (tertile 1: ≤ 249, n = 830; tertile 2: 249-296, n = 824; tertile 3: ≥ 296, n = 819), for those with radial access only (n = 1153) (tertile 1: ≤ 272, n = 393; tertile 2: 272-318, n = 382; tertile 3: ≥ 318, n = 378), and for those with femoral access only (n = 1320) (tertile 1: ≤ 228, n = 441; tertile 2: 228-275, n = 448; tertile 3: ≥ 275, n = 431).

Outcomes assessed were ischemic MACE and bleeding complications that occurred between the procedure and hospital discharge and all-cause mortality up to 1-year post-PCI. Ischemic MACE was defined as a composite of periprocedural myocardial infarction (MI) and stroke. Definitions for blood product requirement, access site bleeding, retroperitoneal bleeding, gastrointestinal bleeding, other bleeding, PCI-related MI, and stroke are from the NCDR CathPCI Registry data dictionary. The need for blood products was defined as packed blood cell transfusion between the start of the procedure and within 72 hours after the procedure or prior to discharge. Access site bleeding was defined as observed and documented in the medical record associated with a hemoglobin drop greater than or equal to 3 g/dL or requiring blood transfusion. Retroperitoneal bleeding and gastrointestinal bleeding were defined as observed and documented in the medical record to be associated with a hemoglobin drop greater than or equal to 3 g/dL, requiring blood transfusion, or requiring procedural intervention to achieve hemostasis. Other bleeding was defined as a confirmed bleeding event not available for selection within the registry that was observed and documented in the medical record to be associated with a hemoglobin drop greater than or equal to 3 g/dL, blood transfusion, or requiring procedural intervention to achieve hemostasis. Any bleeding complication was defined as a composite of all individual bleeding outcomes including the need for blood products. Preprocedure hemoglobin was defined as the last value within 30 days prior to the procedure and postprocedure hemoglobin was defined as the lowest value within 72 hours after the procedure.

PCI–related MI was defined by troponin elevation greater than 5 times the 99th percentile in patients with normal baseline values or a rise in troponin greater than 20% if baseline values were elevated. In addition, symptoms suggestive of myocardial ischemia, new thrombotic electrocardiographic changes, angiographic findings consistent with a procedural complication, imaging evidence of loss of viable myocardium, or new regional wall motion abnormality were required.^20^ Stroke was defined as an acute episode of focal or global cerebral or spinal dysfunction caused by intraparenchymal, intraventricular, or subarachnoid hemorrhage or caused by brain, spinal cord, or retinal vascular injury as a result of infarction of central nervous system tissue.

### Statistical methods

Categorical data are presented as percentages and continuous data are presented as mean ± SD. One-way analysis of variance, χ^2^, and Fisher exact tests were used as appropriate to compare patient characteristics, procedural characteristics, and complications between tertiles. Multivariable linear regression with forward and backward procedures was used to assess correlates of peak ACT. Mortality was evaluated with time-to-event analysis using Kaplan-Meier curves with log-rank statistics to assess all-cause mortality at 30 days, 6 months, and 1 year by peak ACT tertile. Wald χ^2^ testing was used to assess the goodness-of-fit. Landmark Kaplan-Meier analysis for all-cause mortality was conducted from 30 days to 1 year by peak ACT tertile. A univariate Cox proportional hazards regression model was used to estimate hazard ratios for all-cause mortality between peak ACT tertiles. Adjusted mortality analysis using baseline patient demographics, clinical presentation, and procedural characteristics determined as confounders a priori was conducted with a multivariable Cox proportional hazards regression model. Variables entered into the Cox proportional hazards model included age, sex, BMI, smoking status, hypertension, hyperlipidemia, diabetes, dialysis, ACS, prior MI, prior PCI, cardiogenic shock, and cardiac arrest. Analyses were performed using IBM SPSS Statistics for MacOS, version 29 (IBM Corp), and a 2-sided *P* value <.05 was considered statistically significant.

## Results

### Patient characteristics

A total of 2473 patients were included in the study with characteristics as noted in Table 1. The overall mean patient age was 65 ± 12 years, 26% were female, 59.7% were of White race, and 22.7% were of Hispanic ethnicity. Hypertension (81.6%), hyperlipidemia (78.4%), and diabetes (43.8%) were the most common comorbidities and 33.7% of patients had prior PCI.

**Table 1:** Patient characteristics.

	All patients (N = 2473)	Peak ACT tertile			*P* value	Access site		*P* value
		Tertile 1 (n = 830) ≤249	Tertile 2 (n = 824) 249-296	Tertile 3 (n = 819) ≥296		Femoral (n = 1320)	Radial (n = 1153)	
Age, y	65 ± 12	62 ± 12	65 ± 12	67 ± 12	<.001	63 ± 13	66 ± 12	<.001
Body mass index, kg/m^2^	28.67 ± 5.61	29.04 ± 5.60	28.85 ± 5.58	28.13 ± 5.63	.002	28.37 ± 5.48	29.03 ± 5.74	.004
Female sex	643 (26.0%)	183 (22.0%)	219 (26.6%)	241 (29.4%)	.008	356 (27.0%)	287 (24.9%)	.29
Smoker	1029 (41.6%)	350 (42.2%)	342 (41.5%)	337 (41.1%)	.913	514 (38.9%)	515 (44.7%)	<.001
Hypertension	2018 (81.6%)	629 (75.8%)	696 (84.5%)	693 (84.6%)	<.001	1058 (80.2%)	960 (83.3%)	.05
Dyslipidemia	1938 (78.4%)	611 (73.6%)	652 (79.1%)	675 (82.4%)	<.001	1025 (77.7%)	913 (79.2%)	.36
Family Hx of premature CAD	356 (14.4%)	165 (19.9%)	101 (12.3%)	90 (11.0%)	<.001	247 (18.7%)	109 (9.5%)	<.001
Prior MI	629 (25.4%)	180 (21.7%)	217 (26.3%)	232 (28.3%)	.006	365 (27.7%)	264 (22.9%)	.01
Congestive heart failure	730 (29.5%)	148 (17.8%)	267 (32.4%)	315 (38.5%)	<.001	302 (22.9%)	428 (37.1%)	<.001
Prior PCI	834 (33.7%)	230 (27.7%)	299 (36.3%)	305 (37.2%)	<.001	438 (33.2%)	396 (34.3%)	.54
Dialysis	269 (10.9%)	65 (7.8%)	120 (14.6%)	84 (10.3%)	<.001	217 (16.4%)	52 (4.5%)	<.001
Cerebrovascular disease	255 (10.3%)	62 (7.5%)	87 (10.6%)	106 (12.9%)	.001	148 (11.2%)	107 (9.3%)	.12
Peripheral vascular disease	262 (10.6%)	72 (8.7%)	95 (11.5%)	95 (11.6%)	.088	160 (12.1%)	102 (8.8%)	.01
Chronic lung disease	196 (7.9%)	64 (7.7%)	63 (7.6%)	69 (8.4%)	.81	133 (10.1%)	63 (5.5%)	<.001
Diabetes	1084 (43.8%)	327 (39.4%)	371 (45.0%)	386 (47.1%)	.005	573 (43.4%)	511 (44.3%)	.65
Race/ethnicity					<.001			<.001
American Indian/Alaskan Native	33 (1.3%)	14 (1.7%)	12 (1.5%)	7 (0.9%)	–	30 (2.3%)	3 (0.3%)	–
Asian	89 (3.6%)	40 (4.8%)	27 (3.3%)	22 (2.7%)	–	69 (5.2%)	20 (1.7%)	–
Black	191 (7.7%)	66 (8.0%)	40 (4.9%)	85 (10.4%)	–	82 (6.2%)	109 (9.5%)	–
Hispanic	562 (22.7%)	163 (19.6%)	217 (26.3%)	182 (22.2%)	–	296 (22.4%)	266 (23.1%)	–
Other/unknown	117 (4.7%)	32 (3.9%)	43 (5.2%)	42 (5.1%)	–	49 (3.7%)	68 (5.9%)	–
White	1476 (59.7%)	515 (62.0%)	482 (58.5%)	479 (58.5%)	–	789 (59.8%)	687 (59.6%)	–

Values are the mean ± SD for continuous variables and n (%) for categorical variables.

ACT, activated clotting time; CAD, coronary artery disease; MI, myocardial infarction; PCI, percutaneous coronary intervention.

Higher peak ACT was associated with older age, lower BMI, and female sex. Patients with higher peak ACT were more likely to have diabetes, cerebrovascular disease, congestive heart failure, hypertension, hyperlipidemia, and prior PCI. Older age, female sex, congestive heart failure, dialysis dependence, and prior MI correlated to higher peak ACT values in multivariable linear regression (Supplemental Table S1). Patients who self-identified as Asian or American Indian/Alaskan Native were more likely to be in lower peak ACT tertiles.

Patients with femoral access were more likely to have prior MI, be on dialysis, and have chronic lung disease or peripheral vascular disease. Patients who self-identified as Asian or American Indian/Alaskan Native were more likely to have femoral access. Patients with radial access were older, had higher BMI, and were more likely to have hypertension and congestive heart failure.

### Procedural characteristics

Procedural characteristics are shown in Table 2. In the entire population, 25.4% of patients presented with non–ST-elevation myocardial infarction, 21.8% presented with STEMI, 33.8% presented with stable angina, 4.9% presented with unstable angina, 3.4% had atypical chest pain, and 10.7% had another indication for PCI. Of these patients, 3.2% had cardiogenic shock, 2.2% had cardiac arrest, and 8.4% required mechanical circulatory support. Femoral access was used in 53.4% of patients and radial access was used in 46.6% of patients. Ultrasound-guided femoral access was implemented in all cases after June 2018 and utilized in 21.6% of the TF cases overall.

**Table 2:** Procedural characteristics.

	Total (N = 2473)	Peak ACT tertile			*P* value	Access site		*P* value
		Tertile 1 (n = 830) ≤249	Tertile 2 (n = 824) 249-296	Tertile 3 (n = 819) ≥296		Femoral (n = 1320)	Radial (n = 1153)	
Presentation								
STEMI	539 (21.8%)	287 (34.6%)	146 (17.7%)	106 (12.9%)	<.001	383 (29.0%)	156 (13.5%)	<.001
NSTEMI	627 (25.4%)	204 (24.6%)	227 (27.5%)	196 (23.9%)	–	306 (23.2%)	321 (27.8%)	–
Unstable angina	122 (4.9%)	71 (8.6%)	30 (3.6%)	21 (2.6%)	–	113 (8.6%)	9 (0.8%)	–
Stable angina	835 (33.8%)	204 (24.6%)	292 (35.4%)	339 (41.4%)	–	380 (28.8%)	455 (39.5%)	–
Atypical chest Pain	85 (3.4%)	5 (0.6%)	3 (0.4%)	3 (0.4%)	–	11 (0.8%)	0 (0%)	–
Other	265 (10.7%)	59 (7.1%)	126 (15.3%)	154 (18.8%)	–	127 (9.6%)	212 (18.3%)	–
Cardiogenic shock	79 (3.2%)	49 (5.9%)	17 (2.1%)	13 (1.6%)	<.001	75 (5.7%)	4 (0.3%)	<.001
Cardiac arrest	54 (2.2%)	28 (3.4%)	14 (1.7%)	12 (1.4%)	0.015	43 (3.3%)	11 (1.0%)	<.001
LMWH	81 (3.3%)	51 (6.1%)	14 (1.7%)	16 (2.0%)	<.001	66 (5.0%)	15 (1.3%)	<.001
Aspirin	2381 (96.3%)	808 (97.3%)	790 (95.9%)	783 (95.6%)	0.130	1285 (97.3%)	1096 (95.1%)	0.003
GPI	702 (28.4%)	501 (60.4%)	136 (16.5%)	65 (7.9%)	<.001	656 (49.7%)	46 (4.0%)	<.001
Any P2Y12 inhibitor	2316 (93.7%)	771 (92.9%)	780 (94.7%)	765 (93.4%)	0.317	1224 (92.7%)	1092 (94.7%)	0.044
Clopidogrel	1702 (68.8%)	599 (72.2%)	543 (65.9%)	560 (68.4%)	0.021	997 (75.5%)	705 (61.1%)	<.001
Prasugrel	177 (7.2%)	104 (12.5%)	40 (4.9%)	33 (4.0%)	<.001	146 (11.1%)	31 (2.7%)	<.001
Ticagrelor	502 (20.3%)	97 (11.7%)	213 (25.8%)	192 (23.4%)	<.001	124 (9.4%)	378 (32.8%)	<.001
Cangrelor	262 (10.6%)	47 (5.7%)	106 (12.9%)	109 (13.3%)	<.001	55 (4.2%)	207 (18%)	<.001
Thrombolytics	73 (3.0%)	29 (3.5%)	27 (3.3%)	17 (2.1%)	0.187	31 (2.3%)	42 (3.6%)	0.058
Angiographic findings								
1VD not LM	2028 (82.0%)	701 (84.5%)	673 (81.7%)	654 (79.9%)	<.001	1077 (81.6%)	951 (82.5%)	0.659
2VD not LM	245 (9.9%)	60 (7.2%)	93 (11.3%)	92 (11.2%)	–	131 (9.9%)	114 (9.9%)	–
3VD not LM	28 (1.1%)	3 (0.4%)	12 (1.5%)	13 (1.6%)	–	15 (1.1%)	13 (1.1%)	–
LM + 1VD	25 (1.0%)	1 (0.1%)	7 (0.8%)	17 (2.1%)	–	17 (1.3%)	8 (0.7%)	–
LM + 2VD	10 (0.4%)	3 (0.4%)	3 (0.4%)	4 (0.5%)	–	7 (0.5%)	3 (0.3%)	–
LM + 3VD	2 (0.1%)	1 (0.1%)	1 (0.1%)	0 (0.0%)	–	1 (0.1%)	1 (0.1%)	–
LM	20 (0.8%)	2 (0.2%)	6 (0.7%)	12 (1.5%)	–	8 (0.6%)	12 (1.0%)	–
Branch vessel	115 (4.7%)	59 (7.1%)	29 (3.5%)	27 (3.3%)	–	64 (4.8%)	51 (4.4%)	–
Mechanical circulatory support	208 (8.4%)	76 (9.2%)	56 (6.8%)	76 (9.3%)	0.123	179 (13.6%)	29 (2.5%)	<0.001
Fluoroscopy time, min	22.92 ± 15.72	19.44 ± 12.41	23.10 ± 15.22	26.24 ± 18.27	<0.001	24.35 ± 18.16	21.28 ± 12.16	<0.001
Contrast volume, mLs	197 ± 97	212 ± 93	189 ± 95	189 ± 101	<0.001	234 ± 99	153 ± 73	<0.001

Values are the mean ± SD for continuous variables and n (%) for categorical variables.

ACT, activated clotting time; GPI, glycoprotein IIb/IIIa inhibitors; LM, left main; LMWH, low molecular weight heparin; NSTEMI, non–ST-elevation myocardial infarction; STEMI, ST-elevation myocardial infarction; VD, vessel disease.

All patients received UFH, and other medications administered 24 hours prior to the PCI procedure to the end of the PCI procedure included aspirin (96.3%), GPI (28.4%), clopidogrel (68.8%), ticagrelor (20.3%), and cangrelor (10.6%). The majority of patients had non-left main 1 vessel coronary artery disease (82.0%) followed by non-left main 2 vessel coronary artery disease (9.9%). Patients in higher peak ACT tertiles were more likely to have stable angina, angiographic left main involvement, longer fluoroscopy time, and more likely to have received ticagrelor or cangrelor. Patients in lower peak ACT tertiles were more likely to have STEMI and to have received GPI, clopidogrel, or prasugrel.

Patients with femoral access were more likely to have had STEMI and had longer fluoroscopy time. There was greater use of cangrelor and ticagrelor with radial access and greater use of prasugrel, clopidogrel, and GPI with femoral access.

### Bleeding complications and ischemic MACE by peak ACT tertile

Of the 2473 patients, 218 patients (8.8%) had a complication (Supplemental Table S2). Ischemic MACE occurred in 44 patients (1.8%), periprocedural MI in 32 patients (1.3%), and a cerebrovascular accident in 13 patients (0.5%). Bleeding complications occurred in 174 patients (7%), with 57 patients (2.3%) having an access site bleed, 11 patients (0.4%) having a retroperitoneal bleed, 22 patients (0.9%) having a gastrointestinal bleed, and 87 patients (3.5%) with another bleed. A total of 124 patients (5%) required blood products. Of the 174 patients who had bleeding events, 30 patients had BARC 2 bleeding and 144 had BARC 3 bleeding or above. There was no difference in bleeding complications or ischemic MACE by peak ACT tertile in the entire population.

**Figure 2:**
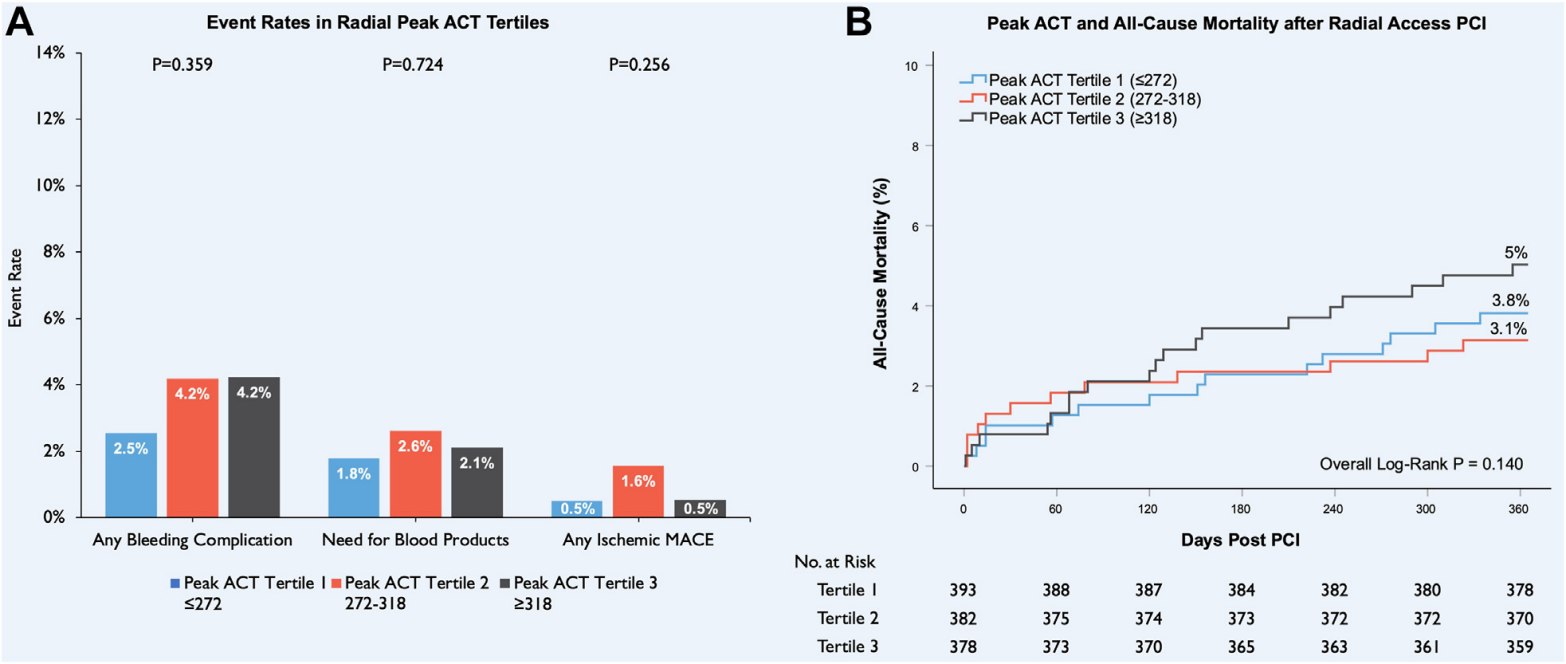
**Impact of peak procedural activated clotting time (ACT) on procedural complications and all-cause mortality after radial access percutaneous coronary intervention (PCI).** (**A**) Procedural complication event rates with radial PCI by peak ACT tertiles. Bar graph of event rates for radial peak ACT tertiles. (**B**) Kaplan-Meier survival estimates for all-cause mortality at 1-year post PCI with radial access. The plot of 1 − survival for radial peak ACT tertiles with the number at risk. MACE, major adverse cardiovascular events.

Among patients with radial access, 42 patients (3.6%) had a bleeding complication, 14 patients (1.2%) had bleeding at the percutaneous entry site, and 10 patients (0.9%) had an ischemic MACE (Table 3). There was no difference in ischemic MACE or bleeding complications by peak ACT tertile in radial access PCI (Figure 2A).

**Table 3:** Outcomes by peak ACT tertile.

Outcome	Radial access			*P* value	Femoral access			*P* value
	ACT tertile				ACT tertile			
	Tertile 1 (n = 393) ≤272	Tertile 2 (n = 382) 272-318	Tertile 3 (n = 378) ≥318		Tertile 1 (n = 441) ≤228	Tertile 2 (n = 448) 228-275	Tertile 3 (n = 431) ≥275	
Any bleeding Complication	10 (2.5%)	16 (4.2%)	16 (4.2%)	.359	33 (7.5%)	44 (9.8%)	55 (12.8%)	.034
Blood products	7 (1.8%)	10 (2.6%)	8 (2.1%)	.724	24 (5.4%)	30 (6.7%)	45 (10.4%)	.014
Access site bleed	1 (0.3%)	7 (1.8%)	6 (1.6%)	.078	8 (1.8%)	15 (3.3%)	20 (4.6%)	.063
Retroperitoneal bleed	0 (0.0%)	0 (0.0%)	1 (0.3%)	.328	5 (1.1%)	2 (0.4%)	3 (0.7%)	.491
Gastrointestinal bleed	4 (1.0%)	2 (0.5%)	3 (0.8%)	.847	3 (0.7%)	4 (0.9%)	6 (1.4%)	.538
Other bleed	5 (1.3%)	7 (1.8%)	6 (1.6%)	.820	20 (4.5%)	23 (5.1%)	26 (6.0%)	.607
Any ischemic MACE	2 (0.5%)	6 (1.6%)	2 (0.5%)	.256	11 (2.5%)	16 (3.6%)	7 (1.6%)	.189
Periprocedural MI	1 (0.3%)	3 (0.8%)	1 (0.3%)	.542	8 (1.8%)	13 (2.9%)	6 (1.4%)	.262
CVA	1 (0.3%)	3 (0.8%)	1 (0.3%)	.542	4 (0.9%)	3 (0.7%)	1 (0.2%)	.505
30-d mortality	4 (1.0%)	5 (1.3%)	3 (0.8%)	.826	4 (0.9%)	12 (2.7%)	23 (5.3%)	<.001
6-mo mortality	9 (2.3%)	9 (2.4%)	13 (3.4%)	.545	9 (2.0%)	18 (4.0%)	27 (6.3%)	.007
1-y mortality	15 (3.8%)	12 (3.1%)	19 (5.0%)	.405	12 (2.7%)	27 (6.0%)	39 (9.0%)	<.001

Values are the number and percentage of patients.

ACT, activated clotting time; CVA, cerebrovascular accident; MACE, major adverse cardiovascular event; MI, myocardial infarction.

Among patients with femoral access, 132 patients (10%) had a bleeding complication, 99 patients (7.5%) required blood products, 43 patients (3.3%) had bleeding at the percutaneous entry site, 10 patients (0.8%) had retroperitoneal bleeding, 13 patients (1.0%) had gastrointestinal bleeding, and 69 patients (5.2%) had other bleeding (Table 3). Patients who had bleeding complications with femoral access had a greater drop in hemoglobin (3.3 ± 2.0 g/dL vs 2.0 ± 2.1 g/dL; *P* < .001) and were more likely to have BARC 3 bleeding (90.2% vs 59.5%; *P* < .001) compared to patients who had bleeding complications with radial access. There was a higher bleeding complication rate in the third peak ACT tertile compared to the second and first peak ACT tertile (12.8% vs 9.8% vs 7.5%; *P* = .034) and a higher need for blood products (10.4% vs 6.7% vs 5.4%; *P* = .014). TF access patients in the third peak ACT tertile were also more likely to have BARC 3 bleeding (11.8% vs 8.0% vs 7.3%; *P* = .042). There was a trend toward higher rates of access site bleeding in the third peak ACT tertile (4.6% vs 3.3% vs 1.8%; *P* = .063), but this did not reach statistical significance. Ischemic MACE occurred in 34 patients (2.6%). There was no significant difference in ischemic MACE between femoral peak ACT tertiles (Central Illustration A).

**Central Illustration:**
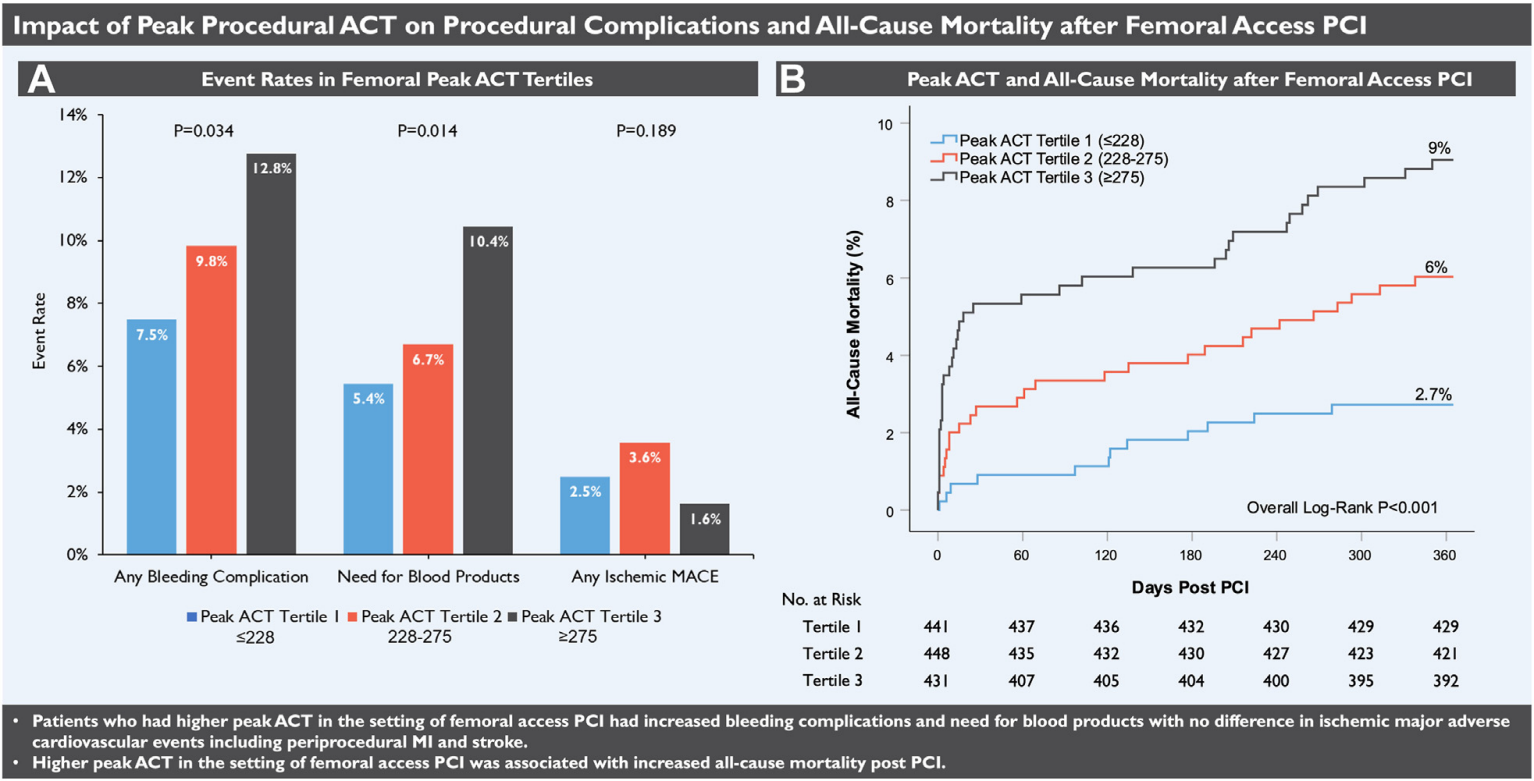
(A) Procedural complication event rates with femoral percutaneous coronary intervention (PCI) by peak activated clotting time (ACT) tertiles. Bar graph of event rates for femoral peak ACT tertiles. (B) Kaplan-Meier survival estimates for all-cause mortality at 1-year post-PCI with femoral access. The plot of 1 − survival for femoral peak ACT tertiles with the number at risk. MACE, major adverse cardiovascular events.

### Unadjusted all-cause mortality by peak ACT tertile

In the entire population, there was 2.1% 30-day all-cause mortality, 3.4% 6-month all-cause mortality, and 5% 1-year all-cause mortality (Supplemental Table S2). There was a significantly higher 1-year all-cause mortality in the third peak ACT tertile and second peak ACT tertile compared to the first peak ACT tertile (5.9% vs 5.7% vs 3.5%; *P* = .048) but no difference in 30-day or 6-month all-cause mortality by peak ACT tertile in the entire population.

For patients who had radial access, there was no significant difference in 30-day, 6-month, or 1-year all-cause mortality by peak ACT tertile (Figure 2B).

**Figure 3:**
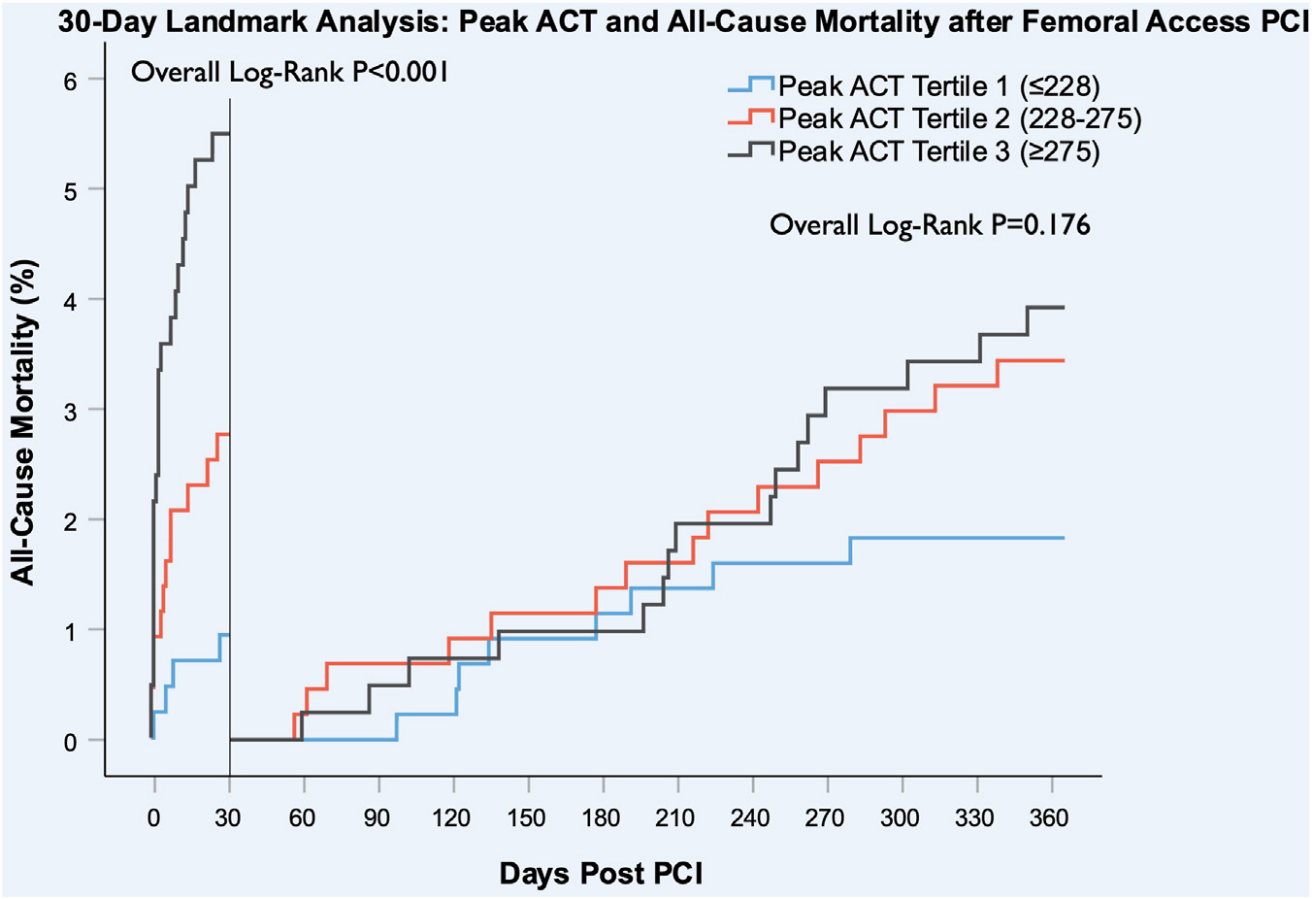
**Thirty-day landmark Kaplan-Meier survival estimates for all-cause mortality at 1-year post-percutaneous coronary intervention (PCI).** Plot of one minus survival for femoral peak activated clotting time (ACT) tertiles.

For patients who had femoral access, patients in the third peak ACT tertile had significantly higher all-cause mortality at 30 days (5.3% vs 2.7% vs 0.9%; overall log-rank *P* < .001) compared to the second and first tertile. There was a statistically significant difference in all-cause mortality at 6 months (6.3% vs 4.0% vs 2.0%; overall log-rank *P* = .007) and 1 year (9.0% vs 6.0% vs 2.7%; overall log-rank *P* < .001) (Central Illustration B), but a 30-day landmark analysis revealed that this difference was nonsignificant after 30 days (3.9% vs 3.4% vs 1.8%; overall log-rank *P* = .176) (Figure 3).

The hazard ratio for 30-day all-cause mortality after femoral access PCI was 6.02 (95% CI, 2.08-17.41; *P* < .001) for the third peak ACT tertile compared to the first peak ACT tertile (Table 4). There was a trend toward higher unadjusted all-cause 30-day mortality for patients in the second femoral peak ACT tertile compared to the first peak ACT tertile (HR, 2.98; 95% CI, 0.96-9.24; *P* = .059), but this did not reach statistical significance.

**Table 4:** Risk of all-cause mortality in femoral peak ACT tertiles.

Outcome	Unadjusted	*P* value	Adjusted^a^	*P* value
	Hazard ratio (95% CI)		Hazard ratio (95% CI)	
30-d all-cause mortality				
Peak ACT tertile 1	1 (reference)		1 (reference)	
Peak ACT tertile 2	2.98 (0.96-9.24)	.059	3.10 (0.97-9.90)	.057
Peak ACT tertile 3	6.02 (2.08-17.41)	<.001	6.64 (2.20-20.02)	<.001
6-mo all-cause mortality				
Peak ACT tertile 1	1 (reference)		1 (reference)	
Peak ACT tertile 2	1.99 (0.90-4.44)	.091	1.78 (0.78-4.07)	.173
Peak ACT tertile 3	3.16 (1.49-6.72)	.003	2.79 (1.25-6.20)	.012
1-y all-cause mortality				
Peak ACT tertile 1	1 (reference)		1 (reference)	
Peak ACT tertile 2	2.25 (1.14-4.44)	.019	1.82 (0.90-3.67)	.094
Peak ACT tertile 3	3.45 (1.81-6.59)	<.001	2.61 (1.32-5.15)	.006
Landmark 30-d to 1-y all-cause mortality				
Peak ACT tertile 1	1 (reference)		1 (reference)	
Peak ACT tertile 2	1.89 (0.80-4.46)	.146	1.20 (0.49-2.92)	.692
Peak ACT tertile 3	2.16 (0.92-5.04)	.076	1.12 (0.46-2.75)	.801

Peak ACT tertile 1, n = 441 ≤ 228; peak ACT tertile 2, n = 434 228-273; peak ACT tertile 3, n = 445 ≥ 273.

ACT, activated clotting time.

a Adjusted for age, sex, body mass index, smoking status, hypertension, hyperlipidemia, diabetes, dialysis, ACS, prior myocardial infarction, prior percutaneous coronary intervention, cardiogenic shock, and cardiac arrest.

### Unadjusted all-cause mortality by peak ACT tertile with or without bleeding complications

Patients who had bleeding complications irrespective of access site had higher mortality at 30 days (12.6% vs 1.3%; *P* < .001), 6 months (15.5% vs 2.5%; *P* < .001), and 1 year (17.8% vs 4.0%; *P* < .001). Of the patients who had femoral access, 132 patients had bleeding complications and 1188 did not have bleeding complications. TF access patients with bleeding complications in the third peak ACT tertile had significantly higher mortality at 30 days (18.2% vs 3%; *P* = .047), 6 months (20.0% vs 3.0%; *P* = .027), and 1 year (23.6% vs 3.0%; *P* = .011) compared to the first peak ACT tertile. TF access patients without bleeding complications in the third peak ACT tertile had significantly higher mortality at 30 days (3.5% vs 0.7%; *P* = .007) and 6 months (6.9% vs 2.7%; *P* = .005), but mortality rates were significantly lower (Supplemental Tables S3 and S4).

There was no difference in mortality by peak ACT tertile among patients who had radial access with or without bleeding complications.

### Adjusted all-cause mortality by peak ACT tertile after femoral access PCI

In Cox multivariable regression adjusting for age, sex, BMI, smoking status, hypertension, hyperlipidemia, diabetes, dialysis, cardiogenic shock, cardiac arrest, acute coronary syndrome, prior MI, and prior PCI, patients in the third peak ACT tertile had significantly higher all-cause mortality at 30 days (HR, 6.64; 95% CI, 2.20-20.02; *P* < .001) after femoral access PCI compared to the first peak ACT tertile (Table 4 and Supplemental Tables S6 and S7). Patients in the third peak ACT tertile also had higher all-cause mortality at 6 months compared to the first tertile (HR, 2.79; 95% CI, 1.25-6.20; *P* = .012) and 1 year (HR, 2.61; 95% CI, 1.32-5.15; *P* = .006) but adjusted 30-day landmark analysis revealed that this difference was driven by events to 30 days. There was a trend toward higher adjusted 30-day all-cause mortality in the second femoral peak ACT tertile compared to the first tertile (HR, 3.10; 95% CI, 0.97-9.90; *P* = .057), but this did not reach statistical significance.

### Outcomes without GPI

Of the 2473 patients, 1771 patients did not receive a GPI. The majority of these patients were treated with TR access (n = 1107) and the rest were treated with TF access (n = 664). Peak ACT tertiles were calculated for TR access without GPI (tertile 1 ≤ 273, n = 372; tertile 2 273-319, n = 374; tertile 3 ≥ 319, n = 361) and TF access without GPI (tertile 1 ≤ 256, n = 225; tertile 2 256-298, n = 219; tertile 3 ≥ 298, n = 220).

There was no difference in bleeding complications or all-cause mortality in patients with radial access who did not receive a GPI (Supplemental Table S5). There was a statistically significant difference in ischemic MACE driven by a higher rate of periprocedural MI in the second peak ACT tertile, but event rates were low.

Among patients with femoral access who did not receive a GPI, there was a higher rate of bleeding complications in the third peak ACT tertile compared to the second and first tertile (15.5% vs 9.1% vs 6.2%; *P* = .005) and a higher need for blood products (13.6% vs 6.8% vs 4.4%; *P* = .001) with no significant difference in ischemic MACE (Table 5). There was a trend toward higher 30-day all-cause mortality in the third peak ACT tertile compared to the second and first tertile (5.0% vs 5.5% vs 1.8%; *P* = .099). There was no difference in bleeding complications, ischemic MACE, or all-cause mortality between peak ACT tertiles in patients who had femoral access and received GPI.

**Table 5:** Outcomes in femoral access without GP IIb/IIIa inhibitors.

Outcome	Femoral access without GPIIb/IIIa (N = 664)Femoral ACT tertile			*P* value tertile 3 vs 1	*P* value tertile 2 vs 1	Overall *P* value
	ACT tertile 1 (n = 225) ≤256	ACT tertile 2 (n = 219) 256-298	ACT tertile 3 (n = 220) ≥298			
Any bleeding Complication	14 (6.2%)	20 (9.1%)	34 (15.5%)	.002	.249	.005
Blood products	10 (4.4%)	15 (6.8%)	30 (13.6%)	<.001	.272	.001
Access site bleed	6 (2.7%)	7 (3.2%)	13 (5.9%)	.091	.741	.169
Retroperitoneal bleed	0 (0.0%)	0 (0.0%)	2 (0.9%)	.244	NA	.218
Gastrointestinal bleed	1 (0.4%)	3 (1.4%)	2 (0.9%)	.620	.367	.460
Other bleed	8 (3.6%)	10 (4.6%)	16 (7.3%)	.083	.589	.185
Any ischemic MACE	3 (1.3%)	7 (3.2%)	4 (1.8%)	.722	.216	.365
Periprocedural MI	2 (0.9%)	6 (2.7%)	3 (1.4%)	.683	.171	.276
CVA	1 (0.4%)	1 (0.5%)	1 (0.5%)	1.000	1.000	1.000
30-d mortality	4 (1.8%)	12 (5.5%)	11 (5.0%)	.060	.036	.099
6-mo mortality	12 (5.3%)	16 (7.3%)	13 (5.9%)	.792	.393	.675
1-y mortality	16 (7.1%)	24 (11.0%)	21 (9.5%)	.352	.157	.364

Values are the number and percentage of patients.

ACT, activated clotting time; CVA, cerebrovascular accident; GP IIb/IIIa, glycoprotein IIb/IIIa; MACE, major adverse cardiovascular event; MI, myocardial infarction.

## Discussion

This study shows that in patients undergoing TF PCI, a therapeutic but high ACT is associated with increased 30-day mortality and major bleeding. This relationship did not exist with TR PCI. Several studies have shown that bleeding after PCI is independently associated with both short-term and long-term mortality which may help explain our findings.^21^, ^22^, ^23^, ^24^ Importantly, with both TR and TF PCI, we found no association between peak procedural ACT tertile and ischemic MACE.

A higher ACT level may have been more important to prevent acute vessel closure in the balloon angioplasty era. With contemporary drug-eluting stents and widespread pretreatment with dual antiplatelet therapy, the rate of periprocedural thrombotic events has declined.^25^^,^^26^ Further, 38.1% of patients in this study received potent P2Y12 inhibition with ticagrelor, cangrelor, or prasugrel in addition to UFH. The observation that ACT tertiles were not related to ischemic MACE suggests that in the contemporary PCI era, the optimal ACT goals may need to be revisited.

The association of a higher ACT in the third tertile with increased mortality and bleeding with TF PCI, even after adjusting for patient and procedure level factors, was predominantly early at 30 days. A 30-day landmark analysis confirmed that this risk of a higher ACT contributing to the adverse events was likely procedure-related. The impact of higher ACT on bleeding complications was independent of GPI use. Moreover, bleeding complications were directly related to mortality in TF PCI, and this risk was exacerbated by high ACT with a trend toward higher access site bleeding.

As complex PCI with and without hemodynamic support continues to require TF access and often large bore access, the various strategies employed to lower access site bleeding require ongoing emphasis. Ultrasound-guided femoral access, use of closure devices and bivalirudin are proven approaches to lower TF access bleeding.^27^, ^28^, ^29^ However, the choice of anticoagulation in chronic total occlusion PCI, atherectomy device use for severely calcified lesions, and with the use of hemodynamic support devices, remains UFH. The current study suggests that in these situations, consideration of lower weight-based UFH dosing and ACT levels might be appropriate. This is particularly relevant with the rise in PCI procedures performed in the frail, aging population who is at increased risk of bleeding.

We found no association between peak procedural ACT tertile and bleeding complications or mortality in patients with radial access undergoing PCI as also shown in previous studies.^12^^,^^13^ Bleeding events with TR PCI were less severe as evidenced by a lower hemoglobin drop and less BARC 3 bleeding compared to TF PCI. The inherently lower bleeding risk with radial access due to smaller vessel size and confined bleeding site likely explains this finding. Furthermore, the lower propensity for occult radial artery bleeding may facilitate earlier detection and management of bleeding complications. Hemostasis can also be achieved more reliably with radial access irrespective of body habitus or vessel calcification. The level of ACT with TR PCI was also not associated with ischemic MACE.

We also observed that patient-level factors including older age, lower BMI, female sex, and dialysis dependence were associated with being in higher peak ACT tertiles highlighting the variable anticoagulant effects of UFH. These subsets of patients are also at increased risk for bleeding complications, especially with femoral access, and are subgroups that should likely receive preferential TR PCI when feasible. There may also be race-based differences in response to UFH as patients who self-identified as Asian, American Indian, or Alaskan Native were more likely to be in lower peak ACT tertiles. This needs to be elucidated in future studies.

### Limitations

The limitations of this study include its retrospective nonrandomized nature using registry data. Only bleeding complications were adjudicated. Further, practice patterns change over time and as this study includes patients treated over 15 years, those changes cannot be accounted for. This was a single-center study conducted on PCI procedures that occurred at an academic medical center in the United States; hence, results may not be generalizable to all institutions. However, the large sample size inclusive of consecutive patients helps obviate some of these limitations.

## Conclusions

This study demonstrates that higher peak ACT with TF access PCI is associated with 30-day and 1-year increased all-cause mortality, bleeding complications, and need for blood products. There is no associated reduction in ischemic MACE, and this association does not exist with TR PCI.

## References

[1] Hirsh J., Anand S.S., Halperin J.L., Fuster V., American Heart Association (2001). Guide to anticoagulant therapy: heparin: a statement for healthcare professionals from the American Heart Association. Circulation.

[2] Lawton J.S., Tamis-Holland J.E., Bangalore S. (2022). 2021 ACC/AHA/SCAI guideline for coronary artery revascularization: a report of the American College of Cardiology/American Heart Association joint committee on clinical practice guidelines. Circulation.

[3] Ferguson J.J., Dougherty K.G., Gaos C.M., Bush H.S., Marsh K.C., Leachman D.R. (1994). Relation between procedural activated coagulation time and outcome after percutaneous transluminal coronary angioplasty. J Am Coll Cardiol.

[4] Narins C.R., Hillegass W.B., Nelson C.L. (1996). Relation between activated clotting time during angioplasty and abrupt closure. Circulation.

[5] Chew D.P., Bhatt D.L., Lincoff A.M. (2001). Defining the optimal activated clotting time during percutaneous coronary intervention: aggregate results from 6 randomized, controlled trials. Circulation.

[6] Pinto D.S., Lorenz D.P., Murphy S.A. (2003). Association of an activated clotting time ≤250 seconds with adverse event rates after percutaneous coronary intervention using tirofiban and heparin (a TACTICS-TIMI 18 substudy). Am J Cardiol.

[7] Montalescot G., Cohen M., Salette G. (2008). Impact of anticoagulation levels on outcomes in patients undergoing elective percutaneous coronary intervention: insights from the STEEPLE trial. Eur Heart J.

[8] Ducrocq G., Jolly S., Mehta S.R. (2015). Activated clotting time and outcomes during percutaneous coronary intervention for non-ST-segment-elevation myocardial infarction: insights from the FUTURA/OASIS-8 trial. Circ Cardiovasc Interv.

[9] Rajpurohit N., Gulati R., Lennon R.J. (2016). Relation of activated clotting times during percutaneous coronary intervention to outcomes. Am J Cardiol.

[10] Tolleson T.R., O’Shea J.C., Bittl J.A. (2003). Relationship between heparin anticoagulation and clinical outcomes in coronary stent intervention: observations from the Esprit trial. J Am Coll Cardiol.

[11] Brener S.J., Moliterno D.J., Lincoff A.M., Steinhubl S.R., Wolski K.E., Topol E.J. (2004). Relationship between activated clotting time and ischemic or hemorrhagic complications: analysis of 4 recent randomized clinical trials of percutaneous coronary intervention. Circulation.

[12] Sharma T., Rylance R., Karlsson S. (2020). Relationship between degree of heparin anticoagulation and clinical outcome in patients receiving potent P2Y12-inhibitors with no planned glycoprotein IIb/IIIa inhibitor during percutaneous coronary intervention in acute myocardial infarction: a VALIDATE-SWEDEHEART substudy. Eur Heart J Cardiovasc Pharmacother.

[13] Louis D.W., Kennedy K., Lima F.V. (2018). Association between maximal activated clotting time and major bleeding complications during transradial and transfemoral percutaneous coronary intervention. JACC Cardiovasc Interv.

[14] Ashby D.T., Dangas G., Aymong E.A. (2003). Relation between the degree of procedural anticoagulation and complications after coronary stent implantation. Am J Cardiol.

[15] Pollak A.Y., Kobo O.M., Margolis G. (Published online May 6, 2024). The imprecision of measuring activated clotting time (ACT) from the guiding catheter during percutaneous coronary interventions. Cardiovasc Revasc Med.

[16] Rozenman Y., Mehran R., Witzenbichler B. (2013). Relationship between the intensity of heparin anticoagulation and clinical outcomes in patients receiving glycoprotein IIb/IIIa inhibitors during primary percutaneous coronary intervention in acute myocardial infarction. Catheter Cardiovasc Interv.

[17] Bertrand O.F., Rodés-Cabau J., Rinfret S. (2009). Impact of final activated clotting time after transradial coronary stenting with maximal antiplatelet therapy. Am J Cardiol.

[18] Gui Y.Y., Huang F.Y., Huang B.T. (2016). The effect of activated clotting time values for patients undergoing percutaneous coronary intervention: a systematic review and meta-analysis. Thromb Res.

[19] Mottillo S., Filion K.B., Joseph L., Eisenberg M.J. (2017). Defining optimal activated clotting time for percutaneous coronary intervention: a systematic review and Bayesian meta-regression. Catheter Cardiovasc Interv.

[20] Thygesen K., Alpert J.S., Jaffe A.S. (2012). Third universal definition of myocardial infarction. J Am Coll Cardiol.

[21] Généreux P., Giustino G., Witzenbichler B. (2015). Incidence, predictors, and impact of post-discharge bleeding after percutaneous coronary intervention. J Am Coll Cardiol.

[22] Kazi D.S., Leong T.K., Chang T.I., Solomon M.D., Hlatky M.A., Go A.S. (2015). Association of spontaneous bleeding and myocardial infarction with long-term mortality after percutaneous coronary intervention. J Am Coll Cardiol.

[23] Baber U., Dangas G., Chandrasekhar J. (2016). Time-dependent associations between actionable bleeding, coronary thrombotic events, and mortality following percutaneous coronary intervention: results from the PARIS registry. JACC Cardiovasc Interv.

[24] Bergmark B.A., Scirica B.M. (2017). First, do no (irreparable) harm: infarction, bleeding, and subsequent risk of death. The dangers of false equivalency. Eur Heart J.

[25] Stefanini G.G., Byrne R.A., Windecker S., Kastrati A. (2017). State of the art: coronary artery stents – past, present and future. EuroIntervention.

[26] Angiolillo D.J., Galli M., Collet J.-P., Kastrati A., O’Donoghue M.L. (2022). Antiplatelet therapy after percutaneous coronary intervention. EuroIntervention.

[27] Mina G.S., Gobrial G.F., Modi K., Dominic P. (2016). Combined use of bivalirudin and radial access in acute coronary syndromes is not superior to the use of either one separately: meta-analysis of randomized controlled trials. JACC Cardiovasc Interv.

[28] Kreutz R.P., Phookan S., Bahrami H. (2022). Femoral artery closure devices vs manual compression during cardiac catheterization and percutaneous coronary intervention. J Soc Cardiovasc Angiogr Interv.

[29] d’Entremont M.A., Alrashidi S., Seto A.H. (2024). Ultrasound guidance for transfemoral access in coronary procedures: an individual participant-level data metaanalysis from the femoral ultrasound trialist collaboration. EuroIntervention.

