# Supplementary material for: Peak Procedural ACT Is Associated With All-Cause Mortality After Femoral Access PCI
Source: J Soc Cardiovasc Angiogr Interv. 2024 Sep 23;3(12):102387. doi: 10.1016/j.jscai.2024.102387 (PMC11725081; doi:10.1016/j.jscai.2024.102387)
Supplement: Supplemental Tables [file mmc1.pdf]

Supplemental Table S1. Coefficients in Multivariable Linear Regression Model for Peak ACT

**Coefficients In Multivariable Linear Regression Model for Peak ACT R^2=0.091 F=26.36 P<0.001**							
	Unstandardized Coefficients	Std Error	Standardized Coefficients	t	P Value	95% Confidence Interval	
CHF	24.01	3.79	0.18	6.34	<.001	16.58	31.43
Age	0.60	0.12	0.13	4.90	<.001	0.36	0.84
Dialysis	14.77	4.21	0.10	3.51	<.001	6.50	23.03
Sex	-11.19	3.53	-0.09	-3.17	0.002	-18.11	-4.27
Prior MI	7.26	3.48	0.06	2.08	0.037	0.42	14.10

Supplemental Table S2. Outcomes by Peak ACT Tertile

		**Total**			
		**ACT Tertile**			
	**Overall**	**Tertile 1 N=830 ≤249**	**Tertile 2 N=824 249-296**	**Tertile 3 N=819 ≥296**	**P Value**
Any Bleeding	174 (7%)	63 (7.6%)	46 (5.6%)	65 (7.9%)	0.131
Blood Products	124 (5.0%)	44 (5.3%)	30 (3.6%)	50 (6.1%)	0.065
Access Site Bleed	57 (2.3%)	19 (2.3%)	14 (1.7%)	24 (2.9%)	0.251
Retroperitoneal Bleed	11 (0.4%)	7 (0.8%)	0 (0.0%)	4 (0.5%)	0.017
Gastrointestinal Bleed	22 (0.9%)	7 (0.8%)	7 (0.8%)	8 (1.0%)	0.948
Other Bleed	87 (3.5%)	33 (4.0%)	25 (3.0%)	29 (3.5%)	0.582
Any Ischemic MACE	44 (1.8%)	18 (2.2%)	17 (2.1%)	9 (1.1%)	0.195
Periprocedural MI	32 (1.3%)	15 (1.8%)	10 (1.2%)	7 (0.9%)	0.224
CVA	13 (0.5%)	4 (0.5%)	7 (0.8%)	2 (0.2%	0.234
30 Day Mortality	51 (2.1%)	10 (1.2%)	23 (2.8%)	18 (2.2%)	0.072
6 Month Mortality	85 (3.4%)	20 (2.4%)	34 (4.1%)	31 (3.8%)	0.128
1 Year Mortality	124 (5.0%)	29 (3.5%)	47 (5.7%)	48 (5.9%)	0.048

Values are number and percent of patients. ACT = activated clotting time; CVA = cerebrovascular accident; MACE = major adverse cardiovascular event; MI = myocardial infarction.

Supplemental Table S3. Transfemoral Access Mortality Analysis With and Without Bleeding

	**Femoral Without Bleeding Complications N=1,188**					**Femoral With Bleeding Complications N=132**				
	**Tertile 1 N=408 ≤228**	**Tertile 2 N=404 228-275**	**Tertile 3 N=376 ≥275**	**P Value Tertile 3 vs 1**	**P Value Tertile 2 vs 1**	**Tertile 1 N=33 ≤228**	**Tertile 2 N=44 228-275**	**Tertile 3 N=55 ≥275**	**P Value Tertile 3 vs 1**	**P Value Tertile 2 vs 1**
30 Day Mortality	3 (0.7%)	7 (1.7%)	13 (3.5%)	0.007	0.222	1 (3.0%)	5 (11.4%)	10 (18.2%)	0.047	0.230
6 Month Mortality	8 (2.0%)	11 (2.7%)	16 (4.3%)	0.062	0.473	1 (3.0%)	7 (15.9%)	11 (20.0%)	0.027	0.128
1 Year Mortality	11 (2.7%)	19 (4.7%)	26 (6.9%)	0.005	0.130	1 (3.0%)	8 (18.2%)	13 (23.6%)	0.011	0.070

Supplemental Table S4. Characteristics of Transfemoral Access Patients With Bleeding

	Femoral Peak ACT Tertile			
	Tertile 1 N=33 ≤228	Tertile 2 N=44 228-275	Tertile 3 N=55 ≥275	P Value
Age	59 (14)	64 (13)	68 (13)	0.013
BMI	29.77 (5.48)	27.33 (4.94)	27.1 (6.71)	0.096
Female	9 (27.3%)	16 (36.4%)	24 (43.6%)	0.304
Smoker	16 (48.5%)	17 (38.6%)	21 (38.2%)	0.593
Hypertension	25 (75.8%)	34 (77.3%)	42 (76.4%)	0.987
Dyslipidemia	22 (66.7%)	37 (84.1%)	38 (69.1%)	0.144
Family Hx Premature CAD	6 (18.2%)	5 (11.4%)	7 (12.7%)	0.667
Prior MI	8 (24.2%)	14 (31.8%)	17 (30.9%)	0.739
CHF	6 (18.2%)	16 (36.4%)	27 (49.1%)	0.015
Prior PCI	9 (27.3%)	17 (38.6%)	12 (21.8%)	0.181
Dialysis	1 (3.0%)	8 (18.2%)	8 (14.5%)	0.129
Cerebrovascular Disease	2 (6.1%)	8 (18.2%)	6 (10.9%)	0.255
Peripheral Vascular	1 (3.0%)	7 (15.9%)	9 (16.4%)	0.149
Chronic Lung Disease	3 (9.1%)	4 (9.1%)	11 (20.0%)	0.198
Diabetes	12 (36.4%)	19 (43.2%)	24 (43.6%)	0.774
Drop in Hemoglobin	3.87 (1.68)	2.77 (1.43)	3.47 (2.37)	0.039

Values are mean ± standard deviation for continuous variables and number and percent of patients for categorical variables. ACT = activated clotting time; BMI = body mass index; CAD = coronary artery disease; MI = myocardial infarction; CHF = congestive heart failure; PCI = percutaneous coronary intervention.

Supplemental Table S5. Outcomes in Radial Access Without GPIIbIIIa

	Radial Access Without GPIIbIIIa N=1,107			
	Radial ACT Tertile			
	Tertile 1 N=372 ≤273	Tertile 2 N=374 273-319	Tertile 3 N=361 ≥319	P Value
Any Bleeding	9 (2.4%)	15 (4.0%)	16 (4.4%)	0.303
Blood Products	6 (1.6%)	9 (2.4%)	8 (2.2%)	0.731
Access Site Bleed	1 (0.3%)	7 (1.9%)	6 (1.7%)	0.097
Retroperitoneal Bleed	0 (0.0%)	0 (0.0%)	1 (0.3%)	0.326
Gastrointestinal Bleed	4 (1.1%)	1 (0.3%)	3 (0.8%)	0.417
Other Bleed	4 (1.1%)	7 (1.9%)	6 (1.7%)	0.657
Any Ischemic MACE	1 (0.3%)	7 (1.9%)	1 (0.3%)	0.032
Periprocedural MI	0 (0.0%)	4 (1.1%)	0 (0.0%)	0.037
CVA	1 (0.3%)	3 (0.8%)	1 (0.3%)	0.628
30 Day Mortality	4 (1.1%)	5 (1.3%)	3 (0.8%)	0.934
6 Month Mortality	9 (2.4%)	9 (2.4%)	12 (3.3%)	0.682
1 Year Mortality	15 (4.0%)	12 (3.2%)	18 (5.0%)	0.475

Values are number and percent of patients. ACT = activated clotting time; CVA = cerebrovascular accident; GP IIb/IIIa = Glycoprotein IIb/IIIa Inhibitors; MACE = major adverse cardiovascular event; MI = myocardial infarction.

Supplemental Table S6. Collinearity Statistics for Cox Proportional Hazards Model

Collinearity Statistics								
	30 day Mortality		6 month Mortality		1 year Mortality		Landmark 30 day to 1 year Mortality	
	Tolerance	VIF	Tolerance	VIF	Tolerance	VIF	Tolerance	VIF
Peak ACT Tertile	0.89	1.12	0.89	1.12	0.89	1.12	0.89	1.13
Age	0.84	1.19	0.84	1.19	0.84	1.19	0.84	1.19
Sex	0.92	1.08	0.92	1.08	0.92	1.08	0.93	1.08
BMI	0.91	1.10	0.91	1.10	0.91	1.10	0.92	1.09
Smoker	0.93	1.07	0.93	1.07	0.93	1.07	0.94	1.07
Hypertension	0.75	1.34	0.75	1.34	0.75	1.34	0.75	1.34
Hyperlipidemia	0.73	1.38	0.73	1.38	0.73	1.38	0.73	1.38
Dialysis	0.78	1.28	0.78	1.28	0.78	1.28	0.79	1.27
Diabetes	0.77	1.30	0.77	1.30	0.77	1.30	0.77	1.30
ACS	0.84	1.19	0.84	1.19	0.84	1.19	0.84	1.19
Cardiogenic Shock	0.76	1.32	0.76	1.32	0.76	1.32	0.82	1.22
Cardiac Arrest	0.76	1.31	0.76	1.31	0.76	1.31	0.83	1.21
Prior MI	0.68	1.48	0.68	1.48	0.68	1.48	0.68	1.47
Prior PCI	0.65	1.55	0.65	1.55	0.65	1.55	0.65	1.54

Supplemental Table S7. Proportionality of Hazards for Cox Proportional Hazards Model

Cox Proportional Hazards Model																
		30 Day Mortality				6 Month Mortality				1 Year Mortality				Landmark 30 Day to 1 Year Mortality		
	HR	95% CI		P Value	HR	95% CI		P Value	HR	95% CI		P Value	HR	95% CI		P Value
Age	1.04	1.01	1.08	0.011	1.04	1.01	1.06	0.005	1.04	1.02	1.06	<.001	1.04	1.01	1.08	0.006
Sex	1.00	0.50	2.01	0.997	0.84	0.47	1.50	0.558	0.75	0.47	1.21	0.234	0.61	0.31	1.19	0.146
BMI	0.95	0.88	1.02	0.140	0.94	0.88	0.99	0.031	0.97	0.92	1.01	0.170	0.98	0.92	1.04	0.435
Smoker	1.58	0.76	3.32	0.223	1.37	0.74	2.52	0.315	1.30	0.79	2.15	0.305	1.20	0.60	2.42	0.602
Hypertension	1.34	0.52	3.48	0.546	1.53	0.65	3.62	0.335	1.38	0.65	2.94	0.398	1.53	0.41	5.69	0.524
Hyperlipidemia	0.45	0.19	1.09	0.078	0.44	0.21	0.95	0.036	0.45	0.23	0.85	0.014	0.50	0.19	1.33	0.164
Diabetes	1.60	0.72	3.56	0.252	1.41	0.72	2.75	0.314	1.44	0.83	2.50	0.190	1.28	0.58	2.86	0.543
Dialysis	2.33	0.91	5.99	0.080	3.17	1.53	6.58	0.002	3.31	1.86	5.91	<.001	4.27	1.92	9.47	<.001
ACS	2.84	1.15	7.05	0.024	2.43	1.22	4.83	0.012	1.96	1.16	3.32	0.012	1.64	0.84	3.20	0.145
Prior PCI	0.93	0.34	2.51	0.879	1.07	0.49	2.33	0.868	1.34	0.73	2.46	0.339	1.73	0.79	3.80	0.169
Prior MI	0.99	0.37	2.67	0.982	1.13	0.52	2.47	0.759	1.12	0.61	2.06	0.712	1.37	0.64	2.98	0.420
Cardiogenic Shock	7.71	3.09	19.21	<.001	5.11	2.23	11.70	<.001	3.74	1.74	8.04	<.001	0.00	0.00	0.00	0.975
Cardiac Arrest	3.98	1.53	10.32	0.005	3.82	1.55	9.37	0.003	3.43	1.47	8.02	0.004	0.00	0.00	0.00	0.981
Femoral Peak ACT Tertie 2 vs 1	3.10	0.97	9.90	0.057	1.78	0.78	4.07	0.173	1.82	0.90	3.67	0.094	1.20	0.49	2.92	0.692
Femoral Peak ACT Tertile 3 vs	6.64	2.20	20.02	<.001	2.79	1.25	6.20	0.012	2.61	1.32	5.15	0.006	1.12	0.46	2.75	0.801
